# Structure‐Property Correlations in CZTSe Domains within Semiconductor Nanocrystals as Photovoltaic Absorbers

**DOI:** 10.1002/advs.202402154

**Published:** 2024-06-18

**Authors:** Apinya Ngoipala, Huan Ren, Kevin M. Ryan, Matthias Vandichel

**Affiliations:** ^1^ Department of Chemical Sciences and Bernal Institute University of Limerick Limerick V94 TP9X Ireland; ^2^ Department of Biological Sciences National University of Singapore 16 Science Drive 4 Singapore 117543 Singapore

**Keywords:** CZTSe nanocrystals, DFT calculations, material interfaces, structure‐optical property relationships

## Abstract

Semiconductor nanocrystals (NCs) are promising materials for various applications. Two of four recently identified Cu_α_Zn_β_Sn_γ_Se_δ_ (CZTSe) domains demonstrate metallic character, while the other two exhibit semiconductor character. The presence of both metallic and semiconductor domains in one NC can hugely benefit future applications. In contrast to traditional band gap studies in the NC community, this study emphasizes that NC domain interfaces also affect the electronic properties. Specifically, the measured band gap of a tetrapod‐shaped CZTSe NC is demonstrated to originate from two specific domains (tetragonal *I* $\bar{4}$ and monoclinic *P*1*c*1 Cu_2_ZnSnSe_4_). The heterojunction between these two semiconductor domains exhibits a staggered type‐II band alignment, facilitating the separation of photogenerated electron‐hole pairs. Interestingly, tetrapod NCs have the potential to be efficient absorber materials with higher capacitance in photovoltaic applications due to the presence of both semiconductor/semiconductor interfaces and metal/semiconductor “Schottky”‐junctions. For the two photo‐absorbing domains, the calculated absorption spectra yield maximum photon‐absorption coefficients of about 10^5^ cm^−1^ in the visible and UV regions and a theoretical solar power conversion efficiency up to 20.8%. These insights into the structure‐property relationships in CZTSe NCs will guide the design of more efficient advanced optical CZTSe materials for various applications.

## Graphical abstract

Four identified domains within tetrapod‐shaped CZTSe nanocrystals have distinct electronic properties. Interestingly, the 3D periodic structure of only two domains exhibits a band gap. Moreover, the computationally predicted type‐II band alignment between these two semiconductor domains facilitates electron‐hole pair separation and enhances solar power conversion efficiency. These insights enable optimizing the design of next‐generation CZTSe‐based solar cells and optoelectronic arrays.

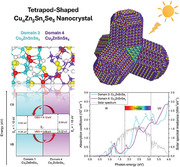

## 1 Introduction

The burgeoning demand for sustainable future energy necessitates the development of efficient semiconductor materials for solar energy conversion. The colloidally synthesized metal chalcogenide semiconductors are highly remarkable as thin‐film solar cell absorber materials due to their tunable bandgap, stability in H_2_O, O_2_ and versatility in device integration.^[^ ^1^ ^]^ One of the most promising thin films for photovoltaic (PV) cells is based on multinary copper‐chalcogenide semiconductors, especially the Cu(In,Ga)(S,Se)_2_ (CIGS) family, which hold a record high efficiency of about 20%.^[^ ^2^ ^]^ Nevertheless, their large‐scale production is limited due to the increasing price of In and Ga. Therefore, increased attention needs to be invested in designing, synthesizing, and developing high‐efficiency and cost‐effective absorbers to replace CIGS. Amongst the multi‐element chalcogenides, the earth‐abundant and non‐toxic Cu_2_ZnSnS_4_ (CZTS) and Cu_2_ZnSnSe_4_ (CZTSe) compounds have been reported as especially promising PV absorber materials with a suitable band gap of 1.0 to 1.5 eV and an intrinsic p‐type conductivity with high absorption coefficients of 10^4^ to 10^5^ cm^−1^.^[^ ^3^ ^]^ However, compared to the well‐established CIGS these compounds still exhibited relatively low solar cell efficiencies until 2023, when a certified power conversion efficiency (PCE) of 13.8% for a kesterite Cu_2_ZnSn(S,Se)_4_ (CZTSSe) solar cell was obtained by tuning the Se partial pressure during the phase evolution.^[^ ^4^ ^]^ The major limitation hindering the high efficiency of CZTS, CZTSe, and CZTSSe solar cells is the large deficit of the open circuit voltage (*V*_OC_‐deficit), which is often associated with structural disorder. Such disorder is manifested through band tail and deep trap states, thereby restricting device performance.^[^ ^5^ ^]^ To reduce the *V*_OC_‐deficit, band gap, defect (doping), and heterojunction interface engineering could aid crystallization and decrease elemental disorder, thus enhancing the PCE.^[^ ^5^, ^6^ ^]^ Previous studies demonstrated the fabrication of CZTSSe solar cells introducting S pulses during the selenization process using graded band gap profiles^[^ ^7^ ^]^ and addressed band engineering by implementing a steep cationic mixing substitution (Sn by Ge) strategy in Cu_2_ZnSn(Sn,Ge)Se_4_ during the formation of precursor layers.^[^ ^8^ ^]^ These devices reached efficiencies above 9% with improved *V*_OC_‐deficit.^[^ ^7^, ^8^ ^]^ Moreover, polytypic non‐stoichiometric CZTSSe nanocrystals (NCs) were synthesized and their phase compositions could be tuned by adjusting the reaction temperature, introducing a novel approach for band‐gap tuning and alignment.^[^ ^9^ ^]^ Recently, the quaternary Cu_α_Zn_β_Sn_γ_Se_δ_ (CZTSe) NCs in their tetrapod‐shaped heterostructures were synthesized using a colloidal dual‐injection method and their physical properties could be tailored by the change of size, shape, and crystal phase.^[^ ^10^ ^]^ This computational study builds on the deciphered NC domains of CZTSe tetrapod NCs from H. Ren et al.^[^ ^10b^ ^]^ and reveals that multiple domains in one NC can have independent and distinct electronic properties. To engineer these PV semiconductors, it is essential to understand the crystal structure‐property correlation via electronic band structure calculations, such as density functional theory (DFT). However, due to the high‐structural and compositional complexity of multi‐element NCs, the unit cells’ stoichiometry ratio and cation substitution are ambiguous. Band structure calculations of this type of NCs typically use random cation substitution to build several possible unit cell models. Therefore, the band gap calculations do not yield conclusive or uniform results.^[^ ^11^ ^]^ Herein, the calculated electronic and optical properties of four deciphered domains within tetrapod‐like CZTSe NCs are presented.^[^ ^10b^ ^]^

## 2 Results and Discussion

**Figure 1:**
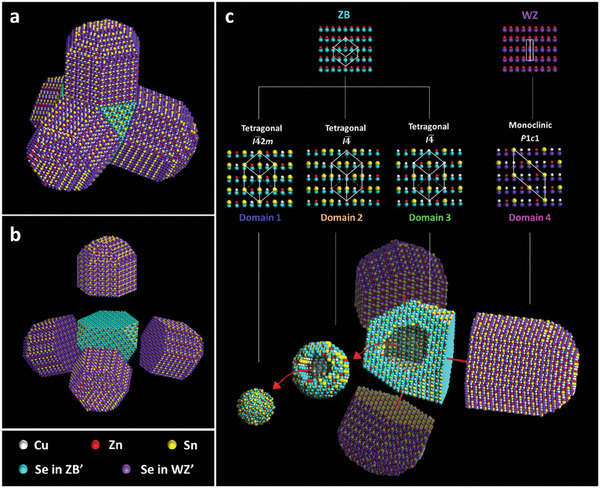
a) Schematic structure model of a Cu_α_Zn_β_Sn_γ_Se_δ_ (CZTSe) tetrapod nanocrystal (NC).^[^ ^10b^ ^]^ b) The exploded view of the schematic model of the CZTSe NC with a zinc blende (ZB)‐derived (core) and four wurtzite (WZ)‐derived (arms) structures. c) The overview of four different domains in the CZTSe NC. Adapted with permission.^[^ ^10b^ ^]^ Copyright 2024, American Chemical Society.

From our previous experimental work, the morphology of the CZTSe tetrapod‐like nanocrystals (NCs) was analyzed through the compilation of transmission and scanning electron microscopy imaging and cation−anion modeling (see experimental methods in Section S1, Supporting Information and ref.[10b] for more details). The CZTSe tetrapod NC (**Figure** **1**a) has a 3D form with a zinc blende (ZB)‐derived (core) and four wurtzite (WZ)‐derived (arms) structures tetrahedrally attached to the ZB core (Figure 1b). The CZTSe NC has Cu, Zn, Sn, and Se elements across the entire NC. However, due to the subsequent introduction of Cu, Zn, and Sn precursors during the synthesis process, four distinct domains with different unit cell structures and chemical stoichiometric ratios are formed (Figure 1c). The three domains from the inner ZB core to the outer layers are all tetragonal domains, with Domain 1 having a *I* $\bar{4}$2*m* space group and structural formula CuZnSn_2_Se_4_, Domain 2 displaying *I* $\bar{4}$ and CuZnSn_2_Se_4_, while Domain 3 has the same space group as Domain 2, i.e., *I* $\bar{4}$, but a different stoichiometry, Cu_2_ZnSnSe_4_. The last WZ‐derived arm has a monoclinic structure, *P*1*c*1, with a chemical ratio the same as Domain 3, Cu_2_ZnSnSe_4_ (Figure 1c).

**Figure 2:**
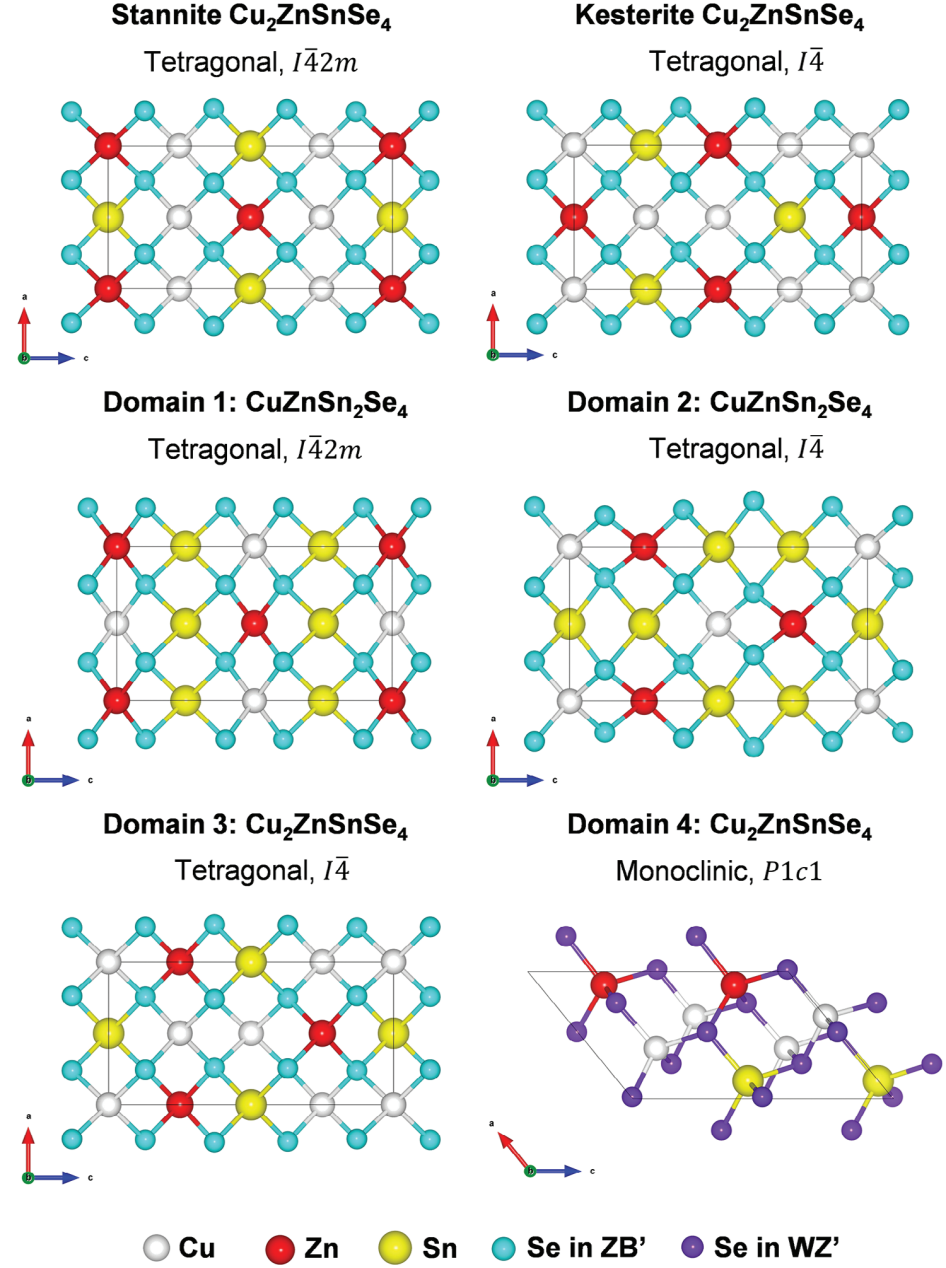
The optimized structures of the unit cells of four domains in tetrapod‐shaped CZTSe nanocrystals obtained by HSE06 functional. The structures of the traditional reported CZTSe (stannite and kesterite) are also presented in the above panel for comparison.

Based on the deciphered atomic arrangements of such four different NC domains obtained in our previous study,^[^ ^10b^ ^]^ the theoretical investigation for the electronic and optical properties of four nanoscaled domains in one nanoparticle entity is made possible for the first time. First, the unit cells of the four domains in CZTSe NC (**Figure** **2**) were optimized using DFT calculations with the hybrid Heyd–Scuseria–Ernzerhof (HSE06) functional. Their calculated and experimental lattice parameters are summarized in **Table** **1**. The computational details and the atomic coordinates of four domains are provided in the Supporting Information, in Section S2 and S3, respectively. HSE06 considers a fraction of exact exchange that is included on top of a semi‐local approximation for the exact exchange–correlation potential.^[^ ^15^ ^]^ As a result, the obtained HSE06‐optimized lattice parameters overestimate the experimental lattice parameters (see Table 1) and this has also been observed in previous theoretical studies employing HSE06.^[^ ^12^, ^16^ ^]^ However, HSE06 has been shown to yield more accurate band gaps for semiconductors, demonstrating a notable improvement over the Perdew, Burke, and Ernzerhof (PBE) functional,^[^ ^17^ ^]^ and was found effective in reproducing the electronic structure of similar materials, e.g., Cu_2_ZnSnSe_4_, Cu_2_ZnSnS_4_, CuInSe_2_, and CuGaSe_2_.^[^ ^18^ ^]^ Therefore, although HSE06 overestimates lattice parameters by about 3.0% to 12.7% compared to experimental values (see Table 1), it provides reliable band gap predictions and consequently the reasonable predicted photovoltaic efficiency as discussed in the next section.

**Table 1:** The experimental^[^ ^10b^ ^]^ and calculated lattice parameters using HSE06 functional of four domains in CZTSe NC. The experimental and calculated lattice parameters of traditional CZTSe materials are also presented for comparison.

Materials	Structural formula	Space group	Calculated values (HSE06 functional)				Experimental values			
			*a* (Å)	*b* (Å)	*c* (Å)	*α, β, γ* (°)	*a* (Å)	*b* (Å)	*c* (Å)	*α, β, γ* (°)
Traditional	Cu_2_ZnSnSe_4_	*I* $\bar{4}$2*m*	5.738	5.738	11.453	90, 90, 90^a)^	5.688	5.688	11.338	90, 90, 90^b)^
Traditional	Cu_2_ZnSnSe_4_	*I* $\bar{4}$	5.732	5.732	11.418	90, 90, 90^a)^	5.693	5.693	11.347	90, 90, 90^c)^
Domain 1	CuZnSn_2_Se_4_	*I* $\bar{4}$2*m*	6.335	6.335	11.279	90, 90, 90	5.795	5.795	10.946	90, 90, 90
Domain 2	CuZnSn_2_Se_4_	*I* $\bar{4}$	6.167	6.167	11.889	90, 90, 90	5.751	5.751	10.550	90, 90, 90
Domain 3	Cu_2_ZnSnSe_4_	*I* $\bar{4}$	5.739	5.739	11.430	90, 90, 90	5.752	5.752	10.449	90, 90, 90
Domain 4	Cu_2_ZnSnSe_4_	*P*1*c*1	6.662	6.992	10.483	90, 129.47, 90	6.733	7.243	10.498	90, 129.90, 90

^a)^ ref. [12];

^b)^ ref. [13];

^c)^ ref. [14]

**Figure 3:**
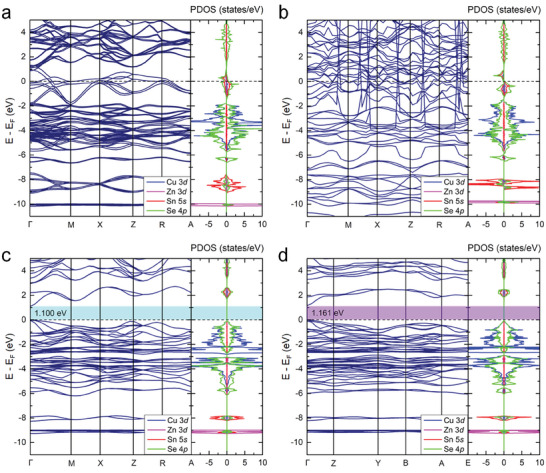
Calculated band structures and partial density of states (PDOS) using HSE06 functional of the four different domains in tetrapod CZTSe NCs; a) Domain 1; b) Domain 2; c) Domain 3; d) Domain 4 (see their atomic structures in Figure 2), where the dashed lines represent the Fermi energy.

It is worth noting that the traditionally reported CZTSe has a tetragonal structure in the stannite (*I* $\bar{4}$2*m*)^[^ ^11^, ^19^ ^]^ or kesterite (*I* $\bar{4}$)^[^ ^20^ ^]^ phase (see Figure 2 in the above panel). Although the deciphered Domains 1 and 2 have a similar space group as the general reported CZTSe (*I* $\bar{4}$2*m* and *I* $\bar{4}$, respectively),^[^ ^3^, ^12^, ^21^ ^]^ their stoichiometry ratios and atomic arrangements are distinct (Figure 2), resulting in different lattice parameters (Table 1). In case of Domain 3, which has the same space group and stoichiometry ratio as the traditional Kesterite CZTSe (*I* $\bar{4}$), the lattice parameters are comparable with those of the traditional ones (Table 1) while the positions of Zn and Sn substitution are different (Figure 2). Accordingly, we expect that the properties of Domain 1, 2, and 3 might be different from those of the obtained conventional CZTSe. In addition, it is intriguing to know whether the identified CZTSe Domain 4 with space group *P*1*c*1 has the potential to be applied in PV devices. In this regard, the band structures and partial density of states (PDOS) of all four domains were calculated using the HSE06 functional to obtain insights into the electronic structure properties of CZTSe tetrapod NC. The calculated band structures of Domain 1 and 2 (**Figure** **3**a,b, respectively) exhibit metallic character as there is no band gap between their valence and conduction bands. According to their PDOS, near the Fermi level, there is overlap between Sn 5*s* and Se 4*p* orbitals with a minor contribution of Cu 3*d* states. Interestingly, the band structures of both Domain 3 and 4 (Figure 3c,d, respectively), demonstrate semiconducting character with direct band gaps of 1.100 and 1.161 eV where the valence band maximum (VBM) and the conduction band minimum (CBM) are located both at the г‐point. These band gap values are in similar range as previous experimental and theoretical studies on traditional CZTSe.^[^ ^3^, ^12^, ^22^ ^]^ In addition, their PDOS, around the Fermi level, reveal that the VBM originates primarily from the hybridization of the Cu 3*d* and Se 4*p* orbitals, offering a conduction pathway for holes in the p‐type semiconductors. Meanwhile, the CBM is predominantly composed of the overlap between Sn 5*s* and Se 4*p* states. Note that for all four domains, the Zn atoms have no contribution to VBM and CBM, whereas their 3*d* states mainly appear in a deeply bonded lower valence band at about −10 eV (for Domain 1 and 2) and −9 eV (for Domain 3 and 4). This can be understood because the atomic orbital energy of Zn 3*d* (−17.3 eV) is much lower than the energy of Se 4*p* (−10.8 eV) compared to that of Cu 3*d* (−13.5 eV).^[^ ^21d^ ^]^ Therefore, Zn 3*d* has a weaker interaction with Se 4*p* and localizes in the lower level of the valence band. The orbital contributions in PDOS for Domain 3 and 4 are consistent with the results of the conventional CZTSe reported in the previous DFT studies.^[^ ^12^, ^21^ ^]^ Our finding suggests that the band gap of the tetrapod CZTSe NC originates from the specific domains and Se coordination in Domain 3 and 4 of the tetrapod structure.

**Figure 4:**
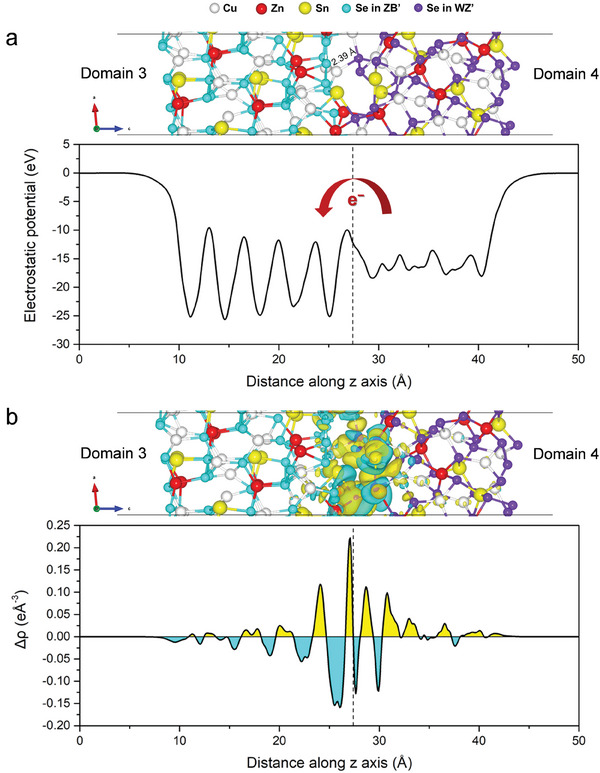
a) Optimized structure of Domain3(112)/Domain4($1\bar{1}0$) interface (upper) at the PBE level of theory and its in‐plane averaged electrostatic potential (lower). b) Isosurfaces of charge density difference of Domain3(112)/Domain4($1\bar{1}0$) interface with an isovalue of 0.0015 eÅ^−3^, where yellow and cyan areas indicate electron accumulation and depletion, respectively (upper) and in‐plane averaged charge density difference of this interface in the *z* direction (lower).

From the analysis of the band structure and PDOS, Domain 1 and 2, which are in the inner core of CZTSe tetrapod NC (see Figure 1) show metallic character while Domain 3 in the outer core and domain 4 in the arm regions of tetrapod NC (Figure 1) demonstrate semiconductor character. As Domain 3 and 4 are connected to each other, it is interesting to explore how the interaction between these domains influences the overall properties of the CZTSe NC. Therefore, we first investigated the energetics of the interaction between Domain 3 and 4 by considering the interface energy and work of adhesion of the Domain 3/Domain 4 heterojunction. Note that the Domain3(112) and Domain4($1\bar{1}0$) facets were experimentally detected from our previous work,^[^ ^10b^ ^]^ we accordingly constructed the surface slabs of Domain3(112) and Domain4($1\bar{1}0$) with different possible terminations and calculated their surface energies (see Supporting Information, Sections S2 and S4 for the computational details and results of these calculations). The most stable surface terminations of the two domain slabs (see Figure S1, Supporting Information) were then taken to model the Domain 3/Domain 4 interface. To obtain the appropriate Domain 3/Domain 4 heterojunction, we considered possible configurations of the interface. As can be seen in Figure S1 (Supporting Information) for the most stable terminations, the Domain3(112) slab consists of two different exposed surfaces which are the Se‐terminated and the Cu/Zn/Sn‐terminated ones, while the Domain4($1\bar{1}0$) slab contains the Se‐terminated surface for both sides. Therefore, we first compared the total energies of the Se‐terminated Domain 3/Se‐terminated Domain 4 and the Cu/Zn/Sn‐terminated Domain 3/Se‐terminated Domain 4 interface models as illustrated in Figure S2 (Supporting Information). Results show that the Cu/Zn/Sn‐terminated Domain 3/Se‐terminated Domain 4 interface has lower energy than the Se‐terminated Domain 3/Se‐terminated Domain 4. Subsequently, different stacking configurations of the Cu/Zn/Sn‐terminated Domain 3/Se‐terminated Domain 4 interface were considered, and their total energies were compared as presented in Figure S3 (Supporting Information). The most stable Domain 3/Domain 4 interface was achieved as displayed in **Figure** **4**a (upper panel), where the shortest distance between these domains is about 2.39 Å, which is Cu(Domain 3)−Se(Domain 4) bond (see Figure 4a (upper panel)). Then, the interface energy (γ_interface_) of this Domain 3/Domain 4 system was calculated using the following equation:^[^ ^23^ ^]^

(1) $$\begin{array}{cc} \gamma_{\mathrm{interface}} & =\frac{1}{2A}\;(E_{\mathrm{Domain}\;3/\mathrm{Domain}\;4}-N_{x}E_{\mathrm{bulk},\;\mathrm{Domain}\;3}\\ & \;-N_{y}E_{\mathrm{bulk},\;\mathrm{Domain}\;4}) \end{array}$$

where *A* represents the surface area of the Domain 3/Domain 4 interface; and E_Domain 3/Domain 4_, E_bulk, Domain 3_, and E_bulk, Domain 4_ are the total energies of the interface system, bulk Domain 3, and bulk Domain 4, respectively; *N_x_* and *N_y_* denote the number of formula units of bulk Domain 3 and Domain 4, corresponding to the number of atoms in the interface system. For the most stable Domain3/Domain 4 interface system, the surface energy (γ_interface_) is 0.092 eVÅ^−2^. This suggests that minimal energy is needed to form the interface from their corresponding bulk. Remarkably, the surface energy of this interface (0.092 eVÅ^−2^) is higher than the individual Domain 3 (0.019 eVÅ^−2^) and Domain 4 (0.024 eVÅ^−2^) surfaces exposing the same surface terminations as in the interface system (see Figure S1, Supporting Information). This implies that there is a thermodynamic driving force for this interface system to separate rather than to stick together. Furthermore, such interface instability might induce interfacial phenomena such as rearrangement of atoms, metal segregation, or formation of additional phase to minimize the surface energy, which will require future computational studies with molecular dynamic simulations to obtain more insight.

Next, the interface bonding strength was investigated by evaluating the interface work of adhesion or binding energy. The work of adhesion (*W*_ad_) is the reversible work required per unit area to separate an interface into two individual surfaces defined as:^[^ ^24^ ^]^

(2) $$W_{\mathrm{ad}}=\left(E_{\mathrm{slab},\;\mathrm{Domain}\;3}+E_{\mathrm{slab},\;\mathrm{Domain}\;4}-E_{\mathrm{Domain}\;3/\mathrm{Domain}\;4}\right)/A$$

where A represents the surface area of the Domain 3/Domain 4 interface; E_slab, Domain 3_, E_slab, Domain 4_, and E_Domain 3/Domain 4_ represent the total energies of Domain 3 slab, Domain 4 slab, and Domain 3/Domain 4 interface, respectively. The W_ad_ was found to be 0.034 eVÅ^−2^, indicating that the interfacial interaction between these two domains is exothermic physical adsorption.

The ideal PV material should maximize the efficiency of charge separation. Upon light irradiation, polarization of uneven distributions between different constituent layers can occur, which leads to the creation of an electric field within the material.^[^ ^25^ ^]^ A substantial built‐in electric field (or internal electric field) is prerequisite to effectively propel charge carriers and separate photogenerated electrons and holes.^[^ ^26^ ^]^ In this regard, we quantified the in‐plane averaged electrostatic potential of the Domain 3/Domain 4 interface (Figure 4a) to determine the internal electric field originated from the heterojunction. As can be seen in Figure 4a (lower panel), a significant potential difference is found at the interface region, indicating the large built‐in electric field pointing from Domain 3 to Domain 4 which promotes the electron migration from Domain 4 to Domain 3. To obtain further insight into the interfacial properties of the Domain 3/Domain 4 system, we calculated the interfacial charge density difference (Δρ) along the *z* direction as depicted in Figure 4b (lower panel) with the isosurfaces of charge density difference (upper panel). The Δρ is defined as:^[^ ^26b^ ^]^

(3) $$\begin{array}{cc} \Delta \rho \left(z\right) & =\int \rho_{\mathrm{Domain}\;3/\mathrm{Domain}\;4}\left(x,\;y,z\right)\;\mathrm{dxdy}\\ & \;-\int \rho_{\mathrm{Domain}\;3}\left(x,\;y,z\right)\;\mathrm{dxdy}\\ & \;-\int \rho_{\mathrm{Domain}\;4}\left(x,\;y,z\right)\;\mathrm{dxdy} \end{array}$$

where ρ_Domain 3/Domain 4_(*x*, *y*, *z*), ρ_Domain 3_(*x*, *y*, *z*), and ρ_Domain 4_(*x*, *y*, *z*) are the charge densities in the Domain 3/Domain 4 interface, Domain 3 slab, and Domain 4 slab at the (*x*,  *y*, *z*) point, respectively. The positive (negative) values of Δρ represent electron charge accumulation (depletion). It is evident from Figure 4b that at the interface region, the charge transfers from Domain 4 to Domain 3, where the electron accumulation (negative charge, yellow isosurface) occurs at the Domain 3 side leaving the positive charge (hole accumulation, cyan isosurface) at the Domain 4 side. Such significant separation of electron‐hole pairs leads to effectively prolonged lifetime of photogenerated carriers in the CZTSe tetrapod NC.^[^ ^27^ ^]^

**Figure 5:**
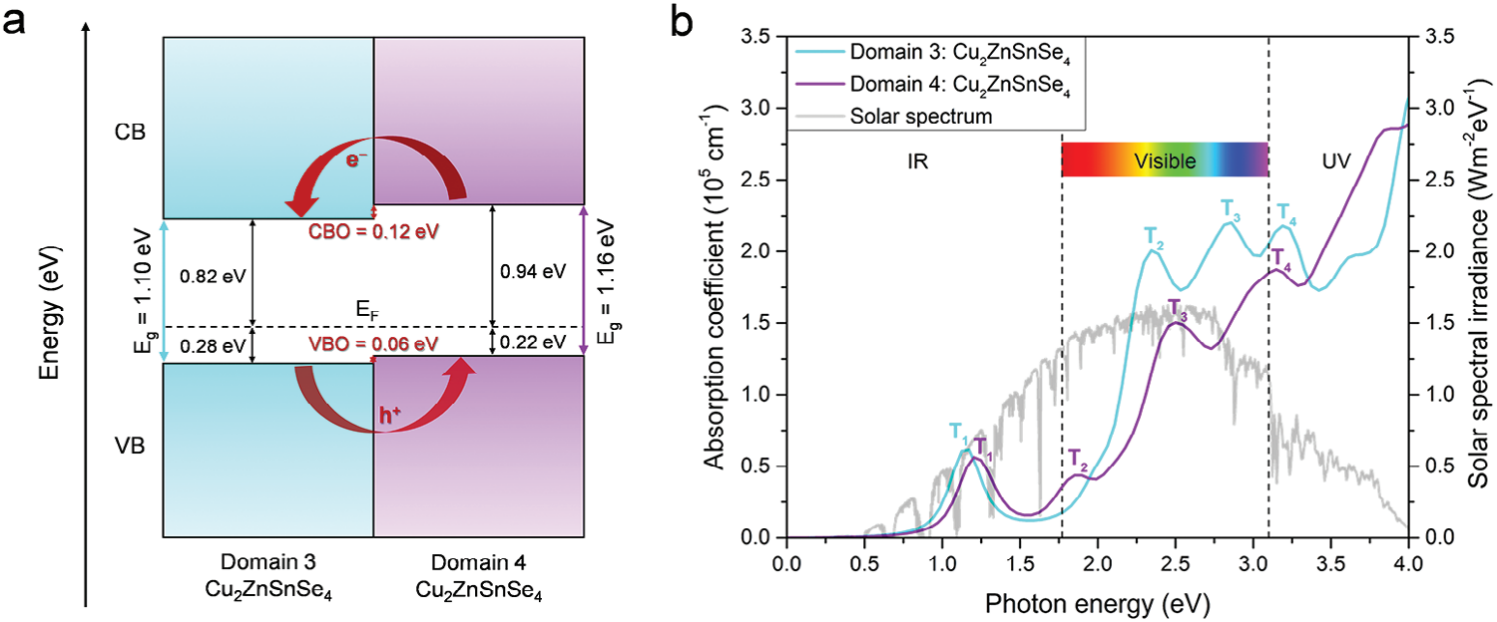
a) Band alignment diagram of the Domain 3/Domain 4 heterojunction from bulk band structure calculations at the HSE06 level of theory, where E_g_, E_F_, VB, CB, VBO, CBO represent band gap energy, Fermi energy, valence band, conduction band, VB offset, and CB offset, respectively. b) Calculated optical absorption spectra of bulk Domain 3 and 4 in CZTSe NC at the HSE06 level of theory, where T_1_, T_2_, T_3_, and T_4_, represent the transition peaks 1, 2, 3, and 4, respectively. The solar spectral irradiance is also plotted which is taken from the AM1.5G solar irradiance data.^[^ ^30^ ^]^

To better understand how the charge carriers can move in this Domain 3/Domain 4 heterojunction, the band alignments of the most stable Domain3(112) and Domain4($1\bar{1}0$) surfaces (see structures in Figure S1, Supporting Information) were first elucidated by determining their positions of VBM, CBM, and work functions. The details for these calculations are provided in the Supporting Information, Section S5 (Supporting Information). It should be noted that the work function is sensitive to the choice of exchange‐correlation functionals,^[^ ^28^ ^]^ which could affect the prediction of band alignments and subsequently the predicted power conversion efficiency. Here, we therefore calculated the in‐plane averaged electrostatic potential of Domain3(112) and Domain4($1\bar{1}0$) surfaces using both PBE and HSE06 functionals to obtain their work functions (see Figure S4, Supporting Information). Results show that HSE06 functional provides higher values of work function (Φ) compared to PBE functional (Φ_PBE_ = 5.207 eV and Φ_HSE06_ = 5.516 eV for Domain 3; Φ_PBE_ = 5.449 eV and Φ_HSE06_ = 5.920 eV for Domain 4). Then, the band alignments of individual Domain3(112) and Domain4($1\bar{1}0$) surfaces were predicted using two different functionals as depicted in Figure S5 (Supporting Information). Note that PBE functional predicts a metallic character (zero band gap) for Domain 3 and 4, we therefore used the band gap values of bulk Domain 3 (1.10 eV) and Domain 4 (1.16 eV) calculated by HSE06 to construct the band alignments for all considered cases. It can be seen from Figure S3 (Supporting Information) that the predicted work function of Domain4($1\bar{1}0$) using HSE06 leads to the lower level of Fermi energy compared to the VBM level, which seems not possible since the Fermi level cannot be lower than the VBM. For the surface slab, its band gap is typically lower than that of the bulk system. This might be one of the reasons for such inconsistency. Furthermore, if the surface is covered by ligands or other species post‐synthesis, it will affect the domain interface. Accordingly, the work function determined from a surface model is extremely crucial for band alignment construction as it is very sensitive to the choice of surface and interface structures. For this reason, we predicted the band alignments of Domain 3 and 4 by determining their positions of VBM, CBM, and Fermi level directly from bulk band structure calculations at the HSE06 level of theory (see Figure S6, Supporting Information) to primarily investigate how the charge transfer can happen in the domain interface. When achieving the band alignments of individual Domains 3 and 4 (Figure S6, Supporting Information), their Fermi levels were aligned to obtain the band alignment diagram of their heterojunction. As illustrated in **Figure** **5**a, the VBM is populated by Domain 4 (donor), while the CBM is distributed on Domain 3 (acceptor), resulting in a staggered type‐II heterojunction, which is desirable for separation of photogenerated electron‐hole pairs in PV application. Within the accuracy of our approximations, the VBM offset (VBO: the VBM difference between Domain 3 and 4) and CBM offset (CBO: the CBM difference between Domain 3 and 4) are predicted to be 0.06 eV and 0.12 eV, respectively. As a result, the photogenerated electrons will flow from Domain 4 to Domain 3 and vice versa for the photogenerated holes (Figure 5a). This finding is consistent with the exploration on the intrinsic built‐in electric field at the Domain 3/Domain 4 interface region (pointing from Domain 3 to Domain 4) and the interfacial charge density difference, as discussed previously in the above section (see Figure 4). Besides the efficient charge separation in tetrapod NC caused by type‐II band offset of semiconductor (Domain 3)/semiconductor (Domain 4) heterojunction, the contact between metallic (Domain 2) and semiconductor (Domain 3) domains forms a depletion region called Schottky junction at Domain 2/Domain 3 interface, offering the band bending necessary for charge separation.^[^ ^29^ ^]^ Therefore, the presence of both metallic and semiconductor domains in tetrapod NCs can be beneficial for PV devices. It remains a challenge to fully describe an entire nanoparticle with different domains, which will require future computational studies with various molecular modelling techniques, e.g., molecular dynamics simulations to study interfaces between different domains and electronic structure calculations.

Having obtained the electronic properties of semiconductor Domains 3 and 4 leads us to examine their optical properties by calculating solar absorption. Here, the frequency‐dependent dielectric functions which consist of real and imaginary parts were examined using the HSE06 functional to obtain the optical absorption coefficient (α), expressed as follows:^[^ ^28^, ^31^ ^]^

(4) $$\alpha \left(\omega \right)=\sqrt{2}\;\frac{\omega }{c}{\left[\sqrt{{\varepsilon_{1}}^{2}\left(\omega \right)+{\varepsilon_{2}}^{2}\left(\omega \right)}-\varepsilon_{1}\left(\omega \right)\right]}^{1/2}$$

where ε_1_ and ε_1_ are the real and imaginary parts of the dielectric function, respectively; *ω* is the light frequency; and c is the speed of the light in vacuum. The details for this calculation are provided in the Section S6 (Supporting Information). As shown in Figure 5b, although Domain 3 and 4 have comparable band gaps, Domain 3 exhibits higher absorption coefficients than Domain 4. Nevertheless, both Domain 3 and 4 possess four optical transition peaks, T_1_, T_2_, T_3_, and T_4_, in the infrared (IR), visible, and ultraviolet (UV) regions. Furthermore, their absorption coefficients yield maximum values of up to 10^5^ cm^−1^ in the visible and UV regions, indicating their excellent potential for PV applications. Note that here, we considered only the direct optical transitions as Domain 3 and 4 are direct band gap semiconductors (see Figure 3c,d). As illustrated in Figure 5b and Figure S7 (Supporting Information), peak T_1_ occurs at around 1.17 eV and 1.20 eV in the IR region for Domain 3 and 4, respectively. These peaks correspond to the electronic transitions between VBM to CBM via Cu‐3*d* → Se‐4*p* and Se‐4*p* → Sn‐5*s*/Se‐4*p*. There are two transition peaks, T_2_ and T_3_ in the visible region for both Domain 3 and 4. For Domain 3, peak T_2_ appears at around 2.34 eV, which arises from the electronic transitions between VBM to CB via Cu‐3*d* → Cu‐3*d/*Zn‐3*d*/Se‐4*p* and Se‐4*p* → Cu‐3*d*/Zn‐3*d*/Sn‐5*s*/Se‐4*p*. The peak T_3_ at around 2.86 eV, also represents a transition between VBM and CB, and can occur via Cu‐3*d* → Cu‐3*d*/Se‐4*p* and Se‐4*p* → Cu‐3*d*/Se‐4*p*. In case of Domain 4, peaks T_2_ and T_3_ emerge at around 1.85 and 2.50 eV, respectively, which is representative for the electron transitions between Cu‐3*d* → Se‐4*p* and Se‐4*p* → Sn‐5*s*/Se‐4*p*. Meanwhile, both domains offer the optical transitions in the UV region, T_4_, at around 3.2 eV, where these transition peaks occur via Cu‐3*d* → Cu‐3*d/*Zn‐3*d*/Se‐4*p* and Se‐4*p* → Cu‐3*d*/Zn‐3*d*/Se‐4*p* for Domain 3, and via Cu‐3*d* → Se‐4*p* and Se‐4*p* → Se‐4*p* for Domain 4. The obtained transitions obey the electronic selection rules in absorption spectroscopy, i.e., the transition is allowed if the change in the total angular momentum (*J*) can be Δ*J*  =  0,   ± 1, where Δ*J*  =  Δ*L* + Δ*S*; *L* is the azimuthal quantum number, and *S* is the spin quantum number.^[^ ^32^ ^]^ The low‐temperature (77 K) photoluminescence (PL) experiments were carried out with a 405 nm laser diode for CZTSe tetrapod NCs and the PL spectra were reported in our previous experimental work (see Figure 5c in ref. [10a]). The PL spectra were shown at the photon energy range from 1.40 eV to 2.80 eV. Five emission peaks were found at 1.78, 2.00, 2.14, 2.22, and 2.29 eV, where peak position at 2.14 eV exhibits the strongest emission intensity. While considering our calculated absorption spectra (Figure 5b), we observed one peak at the photon energy of 2.34 eV for Domain 3 and two peaks at 1.85 eV and 2.50 eV for Domain 4. Our findings reveal that the absorption spectra slightly precedes the PL spectra, implying that the energies at which the tetrapod NC absorbs light are higher (shorter wavelengths) than the energies at which it emits light. This is because the energy lost due to non‐radiative processes (e.g., phonon scattering and thermal relaxation) lowers the energy of the emitted photons relative to the absorbed photons.^[^ ^33^ ^]^ Moreover, the conditions in which PL measurements were conducted such as temperature, pressure, and the presence of other molecules or impurities, can affect the PL spectra, leading to the observed discrepancy between emission and absorption peaks. It should be noted that here we only compared the calculated absorption spectra of Domain 3 and 4 with the experimental PL spectra of the CZTSe tetrapod NCs. To gain more insight, exploring the optical properties of the entire tetrapod NC containing four different domains remains highly challenging, which necessitates future theoretical investigations with the appropriate structural models of NC and large‐scale computational methods.

The obtained type‐II band offset of Domain 3/Domain 4 heterojunction (Figure 5a) and the high solar absorption coefficient of Domain 3 and 4 (Figure 5b) potentially lead to the efficient solar‐to‐electrical energy conversion in this tetrapod NC. This motivates us to theoretically estimate the power conversion efficiency (PCE). The PCE (η) for the Domain 3/Domain 4 interface with a type‐II band alignment which corresponds to the amount of solar energy being converted to electricity can be estimated as the product of *FF* · *V*_OC_ · *J*_SC_ normalized by the incident energy flux, in the limit of 100% external quantum efficiency according to the following equation^[^ ^34^ ^]^

(5) $$\eta \;=\frac{\mathrm{FF}\cdot V_{\mathrm{OC}}\cdot J_{\mathrm{SC}}}{P_{\mathrm{light}}}\;=\frac{0.65\left(E_{g}^{d}-\Delta E_{C}-0.3\right)\int_{E_{g}^{d}}^{\infty }\frac{P\left(\hbar \omega \right)}{\hbar \omega }d\left(\hbar \omega \right)}{\int_{0}^{\infty }P\left(\hbar \omega \right)d\left(\hbar \omega \right)}\;$$

in which *FF* represents the fill factor of 0.65; *V*_OC_ is the estimation of the highest open circuit voltage, which is expressed as the $\left(E_{g}^{d}-\Delta E_{C}-0.3\right)$ term, where $E_{g}^{d}$ is the optical band gap of the donor (Domain 4), ΔE_C_ is the conduction band difference or conduction band offset (see Figure 5a), and the empirical factor of 0.3 accounts for energy conversion kinetics; *J*_SC_ is the short circuit current which is calculated by the integral of the energy dependence of AM1.5 solar energy flux, *P*(ℏ*ω*) in Wm^−2^eV^−1^, weighted by photon energy, ℏ*ω*; *P*_light_ in the denominator is the total solar power of AM1.5 solar energy flux. According to the calculated band gap of Domain 4 (donor) of 1.16 eV and the predicted CBM offset of 0.12 eV, the maximum PCE the Domain 3/Domain 4 interface can deliver, is 20.8%. To date, the reported experimental PCE for kesterite Cu_2_ZnSnSe_4_ thin‐film solar cells is about 11%^[^ ^35^ ^]^ and 14.1% for kesterite Cu_2_ZnSn(S,Se)_4_ solar cells.^[^ ^4^ ^]^ It should be noted that the theoretical estimation of PCE is limited to the calculated values of band gap and CBM offset while the practical PCE includes various factors such as charge carrier transport, excited states and excitonic recombination, etc., which are not considered in this study. Additionally, external factors or environmental factors^[^ ^17^, ^36^ ^]^ such as temperature, illumination, humidity, oxygen exposure, and interaction with ligands or other species post‐synthesis might influence the theoretical predictions, and thereby alter material properties such as conductivity and band gap, affecting the overall energy conversion efficiency of solar cells. However, these fall outside the scope of this work primarily focusing on theoretical predictions within a controlled environment to establish foundational properties. Moreover, the structural, electronic, and optical properties of the whole tetrapod CZTSe NC is a comprehensive aspect for future in‐depth investigation, which goes beyond the scope of the current study. Nevertheless, this study provides the fundamental interpretation of the CZTSe NCs, which comprises different CZTSe phases. Understanding that the electronic and optical properties from CZTSe crystal structure‐dependent electronic and optical properties of CZTSe domains is a substantial understanding for subsequent functional design of semiconductor NCs.

## 3 Conclusions

By computationally studying the deciphered four domains in CZTSe tetrapod‐like nanocrystals (NCs) from our previous experiments using DFT calculations at the HSE06 level of theory, we were able to predict and understand the electronic and optical properties of these domains. The calculated band structures and PDOS reveal that Domain 1 and 2 exhibit the metallic character while Domain 3 and 4 possess the semiconductor character with the direct band gaps of about 1 eV. Interestingly, we unveil that the band gap of the CZTSe semiconductor NC comes from the specific domains, i.e., Domain 3 (tetragonal *I* $\bar{4}$ Cu_2_ZnSnSe_4_) and Domain 4 (monoclinic *P*1*c*1 Cu_2_ZnSnSe_4_) in the structure. Moreover, the Domain 3/Domain 4 heterojunction exhibits the staggered type‐II band alignment, which diminishes the recombination of photogenerated electron‐hole pairs. The absorption coefficients of Domain 3 and 4 yield maximum values up to the order of 10^5^ cm^−1^ in the visible and UV regions, which leads to the efficient solar‐to‐electricity conversion. The theoretical maximum PCE was predicted to be 20.8% for the Domain 3/Domain 4 interface. The obtained results can be crucial for the general explanation on complex semiconductor NC and gives direct guidance toward the design of superior semiconductor NCs for optoelectronic applications. Besides, our computational insights uncover the potential application of specific CZTSe NCs in quantum computing as qubits. By taking advantage of the quantum confinement and semiconductor quantum wells present in tetrapod NCs, it could become possible to encode, manipulate, and store quantum information within their quantized energy levels.

## Conflict of Interest

The authors declare no conflict of interest.

## Supporting information

Supporting Information

## Data Availability

The data that support the findings of this study are available from the corresponding author upon reasonable request.
