# Supplementary material for: Structure‐Property Correlations in CZTSe Domains within Semiconductor Nanocrystals as Photovoltaic Absorbers
Source: Adv Sci (Weinh). 2024 Jun 18;11(31):2402154. doi: 10.1002/advs.202402154 (PMC11336955; doi:10.1002/advs.202402154)
Supplement: Supplementary file 1 — Supporting Information [file ADVS-11-2402154-s001.docx]

**Supporting Information for**

**Structure-Property Correlations in CZTSe Domains within Semiconductor Nanocrystals as Photovoltaic Absorbers**

**Authors:** Apinya Ngoipala**^1^**, Huan Ren**^1,2^**, Kevin M. Ryan**^1^**, Matthias Vandichel***^1^**

**Affiliations:**

1: Department of Chemical Sciences and Bernal Institute, University of Limerick, Limerick, V94 TP9X, Ireland

2: Department of Biological Sciences, National University of Singapore, Singapore, 117543, Singapore

*Corresponding author: [Matthias.Vandichel@ul.ie](mailto:Matthias.Vandichel@ul.ie)

**Contents**

[Section S1: Experimental methods 2](#_Toc167314404)

[Section S2: Computational details 3](#_Toc167314405)

[Section S3: Atomic coordinates of all four domains 5](#_Toc167314406)

[Section S4: Surface energies of Domain 3 and Domain 4, and total energies of different Domain 3/Domain 4 interface configurations 7](#_Toc167314407)

[Section S5: Calculation of band alignment 10](#_Toc167314408)

[Section S6: Calculation of optical absorption coefficient 13](#_Toc167314409)

[References: 15](#_Toc167314410)

# **Section S1: Experimental methods**

Materials:

Copper(I)chloride (Cu(I) Cl >97%), tin(IV) acetate (Sn(Ac)_4_, >99.99%), zinc acetate (Zn(Ac)_2_, >99.99%), oleylamine (OLAM, technical grade, 70%) were purchased from Sigma Aldrich. All chemicals were used as purchased without further processing.

Synthesis protocol:

(Cu(I)Cl (0.25 mmol) and OLAM (10 ml) were added in a three-neck flask and evacuated at 60 °C for 20 minutes. The mother solution was heated to 310 °C in 20 minutes under argon protection. Injection solution 1 contains DPDSe (0.3 mmol), Sn(IV)Ac_4_ (0.125 mmol) and OLAM (2.5 ml). Injection solution 2 contains Zn(Ac)_2_ (0.125 mmol) and OLAM (1.5 ml). Injection solution 1 was injected into the flask at 310 °C and injection solution 2 was injected 7 seconds after injection 1. The solution was allowed to proceed at the same temperature for 15 minutes after the two injections. The reaction was stopped by removing the heating mantle and cooled naturally to 90 °C, then quenched with 30 ml toluene. The product was washed with toluene/isopropanol (3:1) 2 times at 4000 rpm for 2 minutes.

Washing procedure and selective centrifuge separation:

Toluene: isopropyl alcohol (IPA) (1:1) was added in the crude product. Mixture was centrifuged at 4000 rpm for 2 minutes. The upper clear organic solution was discarded. The precipitate was dispersed in 50 ml toluene and left to precipitate naturally for 15 minutes. The upper solution with well-dispersed NCs were collected and washed twice with toluene : IPA (1:1) solution. The final product was dispersed in toluene for further analysis.

Transmission and scanning electron microscopy (TEM and SEM):

The TEM image of CZTSe tetrapod NCs was taken on a JEOL JEM-2011F, operating at an accelerating voltage of 200 kV. The ADF-STEM images and EDS mappings were recorded using an aberration-corrected Titan Themis G2 microscope at 300 kV. The convergence semi-angle of the incident electron probe was approximately 30 mrad, and the ADF images were collected over a detector angle range of 39–200 mrad with the current 89 pA.^[1]^

# **Section S2: Computational details**

First-principles calculations based on a spin-polarized density functional theory (DFT) approach as implemented in the Vienna Ab Initio Simulation Package (VASP 5.4)^[2]^ were carried out on periodic molecular models. The projector augmented-wave method^[3]^ was used to describe the electron-ionic core interactions where Cu 3*d*^10^4*s*^1^, Zn 3*d*^10^4*s*^2^, Sn 5*s*^2^5*p*^2^, and Se 4*s*^2^4*p*^4^ were treated as valence electrons. The generalized gradient approximation with the Perdew, Burke, and Ernzerhof (PBE) exchange-correlation functional generally fails to reproduce the band gaps of materials. Therefore, we employed the hybrid functional of Heyd–Scuseria–Ernzerhof (HSE06)^[4]^, which was found effective in reproducing the electronic structure of similar materials with 30% exact exchange.^[5]^ It was found that the HSE06 functional is superior in terms of localizing valence electrons of transition metals (*e.g.*, those in Cu 3*d* orbitals) more correctly than the (semi)local-density functionals.^[6]^ All structures were optimized with the conjugate gradient method.^[7]^ The Gaussian smearing technique^[8]^ (σ = 0.05 eV) was used during structural relaxations and energies were extrapolated to zero smearing width.

The unit cells of the four domains in CZTSe tetrapod NC were first optimized using the HSE06 functional with a 30% screened Hartree-Fock exchange and a standard Hartree-Fock screening parameter of 0.2. The cutoff energy for the plane-wave basis set was set to 600 eV for all calculations. All systems are structurally cell-optimized until the largest force is smaller than 0.01 eV/Å. Furthermore, the convergence criterion for the electronic self-consistent field (SCF) problem is set to 10^−6^ eV for all optimizations and the symmetry group was preserved throughout all calculations. Subsequently, the HSE06 functional was also applied for the calculations of electronic structures and optical properties for the four optimized domains (see **Figure 2**). When calculating the partial density of states (PDOS), we employed the tetrahedron method with Blöchl corrections and a denser 5×5×3 Monkhorst–Pack^[9]^ k-point grid. The band structures were calculated along high-symmetry points inside the Brillouin zone of the domain unit cells, where the k-path of Γ(0, 0, 0)–M(0.5, 0.5, 0)–X(0, 0.5, 0)–Z(0, 0, 0.5)–R(0.5, 0, 0.5)–A(0.5, 0.5, 0.5) was considered for the tetragonal systems (Domain 1, 2, and 3) and Γ(0, 0, 0)–Z(0, 0.5, 0)–Y(0.5, 0, 0)–B(0, 0, 0.5)–A(0.5, 0, 0.5)–E(0.5, 0.5, 0.5) was considered for the monoclinic system (Domain 4).

To describe the energetics of the interaction between Domain 3 and 4 that show the semiconductor character, Domain3($112$) and Domain4($1\bar{1}0$) surface were considered as the slab models as these facets were experimentally observed from our previous work.^[1]^ The Domain3($112$) and Domain4($1\bar{1}0$) surface slabs with different terminations (see **Figure S1**) were constructed by cleaving their optimized bulk unit cells. A vacuum space of 20 Å along the z direction was added to avoid the artificial interaction between neighboring periodic images. All surface structures were optimized with constant cell volume using the PBE exchange-correlation functional with the cutoff energy of 600 eV and the convergence tolerance of 10^−6^ eV for energy and 0.02 eV/Å for force. For Domain($112$) and Domain4($1\bar{1}0$) surfaces, 3×2×1 and 3×3×1 Monkhorst-Pack k-point grids were employed for structural relaxations, respectively (optimized cell parameters are reported in **Table S1**).

Next, the most stable surface terminations were used to create the Domain 3/Domain 4 interface by placing two different surfaces adjacent to each other and taking the average of their lattice parameters (see **Table S1**) for the interface system size (initial structure before fixed-volume optimization; *a* = 8.887 Å, *b* = 12.251 Å, c = 49.886 Å, *α* = 90°, β = 90°, γ = 103°), including a vacuum space of 20 Å along the z direction, where the initial interfacial distances were set in the range of 2-3 Å. To find the most stable interface, several possible configurations were considered (see **Figure S2 and S3**) and optimized with the cell volume constraint, using the PBE functional with the cutoff energy of 450 eV and the convergence tolerance of 10^−6^ eV for the energy and 0.05 eV/Å for the atomic forces, where the Brillouin zone integration was performed with a single k-point at the Γ-position. The structure that has the lowest total energy was then reoptimized with the cell volume constraint using an un-spin-polarized calculation (to be consistent with the non-magnetic structures of the bulk Domain 3 and 4) with the cutoff energy of 600 eV; the convergence tolerance of 10^−6^ eV for the energy and 0.02 eV/Å for the atomic forces; 3×2×1 Monkhorst-Pack k-point grids, and studied further (see **Figure 4a**). The lattice parameters of this interface and the required cell parameter changes of the individual surfaces to form the interface structure are reported in **Table S1**.

# **Section S3: Atomic coordinates of all four domains**

The unit cells of different domains identified by H. Ren et al.^[1]^ were optimized with the HSE06 functional according to the above computational methodology (Section S2).

1. Domain 1

| Se_1_ | (0.2488, 0.7512, 0.8951) |
| --- | --- |
| Se_2_ | (0.7512, 0.2488, 0.8951) |
| Se_3_ | (0.7512, 0.7512, 0.1049) |
| Se_4_ | (0.2488, 0.2488, 0.1049) |
| Se_5_ | (0.7488, 0.2512, 0.3951) |
| Se_6_ | (0.2512, 0.7488, 0.3951) |
| Se_7_ | (0.2512, 0.2512, 0.6049) |
| Se_8_ | (0.7488, 0.7488, 0.6049) |
| Cu_1_ | (0.0000, 0.0000, 0.5000) |
| Cu_2_ | (0.5000, 0.5000, 0.0000) |
| Zn_1_ | (0.0000, 0.0000, 0.0000) |
| Zn_2_ | (0.5000, 0.5000, 0.5000) |
| Sn_1_ | (0.0000, 0.5000, 0.2500) |
| Sn_2_ | (0.5000, 0.0000, 0.7500) |
| Sn_3_ | (0.0000, 0.5000, 0.7500) |
| Sn_4_ | (0.5000, 0.0000, 0.2500) |

2. Domain 2

| Se_1_ | (0.2020, 0.2601, 0.1180) |
| --- | --- |
| Se_2_ | (0.7971, 0.7399, 0.1180) |
| Se_3_ | (0.2601, 0.7971, 0.8820) |
| Se_4_ | (0.7399, 0.2029, 0.8820) |
| Se_5_ | (0.7029, 0.7601, 0.6180) |
| Se_6_ | (0.2971, 0.2399, 0.6180) |
| Se_7_ | (0.7601, 0.2971, 0.3820) |
| Se_8_ | (0.2399, 0.7029, 0.3820) |
| Cu_1_ | (0.0000, 0.0000, 0.0000) |
| Cu_2_ | (0.0000, 0.0000, 0.0000) |
| Zn_1_ | (0.0000, 0.5000, 0.2500) |
| Zn_2_ | (0.5000, 0.0000, 0.7500) |
| Sn_1_ | (0.0000, 0.5000, 0.7500) |
| Sn_2_ | (0.5000, 0.0000, 0.2500) |
| Sn_3_ | (0.0000, 0.0000, 0.5000) |
| Sn_4_ | (0.5000, 0.5000, 0.0000) |

3. Domain 3

| Se_1_ | (0.2444, 0.2392, 0.1286) |
| --- | --- |
| Se_2_ | (0.7556, 0.7608, 0.1286) |
| Se_3_ | (0.2392, 0.7556, 0.8714) |
| Se_4_ | (0.7608, 0.2444, 0.8714) |
| Se_5_ | (0.7444, 0.7392, 0.6286) |
| Se_6_ | (0.2556, 0.2608, 0.6286) |
| Se_7_ | (0.7392, 0.2556, 0.3714) |
| Se_8_ | (0.2608, 0.7444, 0.3714) |
| Cu_1_ | (0.0000, 0.5000, 0.7500) |
| Cu_2_ | (0.5000, 0.0000, 0.2500) |
| Cu_3_ | (0.0000, 0.0000, 0.0000) |
| Cu_4_ | (0.5000, 0.5000, 0.5000) |
| Zn_1_ | (0.0000, 0.5000, 0.2500) |
| Zn_2_ | (0.5000, 0.0000, 0.7500) |
| Sn_1_ | (0.0000, 0.0000, 0.5000) |
| Sn_2_ | (0.5000, 0.5000, 0.0000) |

4. Domain 4

| Se_1_ | (0.7550, 0.3372, 0.7405) |
| --- | --- |
| Se_2_ | (0.7550, 0.6628, 0.2405) |
| Se_3_ | (0.2771, 0.3380, 0.2590) |
| Se_4_ | (0.2771, 0.6620, 0.7590) |
| Se_5_ | (0.0169, 0.8252, 0.0034) |
| Se_6_ | (0.0169, 0.1748, 0.5034) |
| Se_7_ | (0.5257, 0.8310, 0.4967) |
| Se_8_ | (0.5257, 0.1690, 0.9967) |
| Cu_1_ | (0.6460, 0.1620, 0.4997) |
| Cu_2_ | (0.6460, 0.8380, 0.9997) |
| Cu_3_ | (0.3981, 0.6658, 0.2504) |
| Cu_4_ | (0.3981, 0.3342, 0.7504) |
| Zn_1_ | (0.8952, 0.6701, 0.7506) |
| Zn_2_ | (0.8952, 0.3299, 0.2506) |
| Sn_1_ | (0.1440, 0.1683, 0.9997) |
| Sn_2_ | (0.1440, 0.8317, 0.4997) |

# **Section S4: Surface energies of Domain 3 and Domain 4, and total energies of different Domain 3/Domain 4 interface configurations**

Based on the experimentally observed exposed Domain3($112$) and Domain4($1\bar{1}0$) facets from our previous work,^[1]^ we constructed the surface labs of Domain3($112$) and Domain4($1\bar{1}0$) with different possible terminations (**Figure S1**) and calculated their surface energies ($\gamma$) using the following equation:

$$\gamma=\frac{1}{2A}\left( E_{\mathrm{slab}}-E_{\mathrm{bulk}} \right)$$

where $A$ is the surface area of the slab; the terms $E_{\mathrm{slab}}$ and $E_{\mathrm{bulk}}$ are the total energies of the slab and the bulk containing the same number of atoms as in the slab. The considered surface terminations of Domains 3 and 4 and their calculated surface energies at the PBE level of theory are illustrated in **Figure S1**. Therefore, the most stable surface terminations of Domain 3($112$) and Domain 4($1\bar{1}0$) were taken to model the Domain 3/Domain 4 interface.

**Figure S1** Different surface terminations of Domain3($112$) and Domain4($1\bar{1}0$) and their calculated surface energies ($\gamma$). Different surfaces were modeled by cleaving the optimized structure of their corresponding bulk. Surface energies at the PBE level of theory are given in eV/Å^2^. The calculated cell parameters of these surfaces are listed in **Table S1**.

**Table S1** The optimized cell parameters of the most stable configurations for Domain3($112$) surface, Domain4($1\bar{1}0$) surface (see **Figure S1**), and their fixed-volume optimized interface structure (see **Figure 4a**). The cell changes of individual surfaces are reported relative to the fixed-volume optimized interface structure.

| Materials | *a*  (Å) | *b*  (Å) | *α*  (°) | *β*  (°) | *γ*  (°) | Area  (Å^2^) | Area change  (%) |
| --- | --- | --- | --- | --- | --- | --- | --- |
| Domain3($112$) | 8.116 | 14.019 | 90.00 | 90.00 | 90.00 | 90.99 | 15.09 |
| Domain4($1\bar{1}0$) | 9.658 | 10.483 | 90.00 | 90.00 | 116.01 | 113.77 | −7.95 |
| Domain3($112$)/  Domain4($1\bar{1}0$) interface | 9.289 | 11.632 | 89.35 | 95.15 | 103.29 | 104.73 | - |

**Figure S2** Structures of the Se-terminated Domain 3($112$)/Se-terminated Domain 4($1\bar{1}0$) and the Cu/Zn/Sn-terminated Domain 3($112$)//Se-terminated Domain 4($1\bar{1}0$) interfaces. Their relative energies (E_rel_) at the PBE level of theory are given in eV.

**Figure S3** Different stacking configurations of the Cu/Zn/Sn-terminated Domain 3($112$)/Se-terminated Domain 4($1\bar{1}0$) interface. Their relative energies (E_rel_) at the PBE level of theory are given in eV. The most stable interface (E_rel_ = 0.000 eV) was studied further (see **Figure 4**).

# **Section S5: Calculation of band alignment**

The positions of the valence band maximum ($E_{\mathrm{VBM}}$) and the conduction band minimum ($E_{\mathrm{CBM}}$) of Domain 3 and 4 can be theoretically evaluated using the following empirical equations^[10]^

$E_{\mathrm{VBM}}=\chi-E_{e}+0.5E_{g}$ (*vs* NHE)

$E_{\mathrm{CBM}}=E_{\mathrm{VBM}}-E_{g}$ (*vs* NHE)

where $E_{e}$ is the energy of the free electron with respect to the normal hydrogen electrode (NHE) of 4.5 eV;^[11]^ $E_{g}$ is the band gap energy of the semiconductor which is $E_{g}$ = 1.10 and 1.16 eV for Domain 3 and 4, respectively; and $\chi$ is the electronegativity of the semiconductor which can be obtained by the following equation^[12]^

$$\chi={[{\chi(A)}^{a}{\chi(B)}^{b}{\chi(C)}^{c}{\chi(D)}^{d}]}^{1/(a+b+c+d)}$$

with $a$, $b$, $c$, and $d$ are the number of atoms in the compounds and A, B, C and D the different atoms. The detailed calculation of $\chi$ for Cu_2_ZnSnSe_4_ is provided below, using the atomic ionization energy (EIE)^[13]^ and the electron affinity (EEA)^[13]^ to calculate $\chi$ for each atom.

For Cu: EIE = 7.73 eV and EEA = 1.23 eV

$\chi$(Cu) = ½(7.73 + 1.23) = 4.48 eV

For Zn: EIE = 9.39 eV and EEA = 0.00 eV

$\chi$(Zn) = ½(9.39 + 0.00) = 4.70 eV

For Sn: EIE = 7.34 eV and EEA = 1.20 eV

$\chi$(Sn) = ½(7.34 + 1.20) = 4.27 eV

For Se: EIE = 9.75 eV and EEA = 2.02 eV

$\chi$(Se) = ½(9.75 + 2.02) = 5.89 eV

$\chi$(Cu_2_ZnSnSe_4_) = (4.48^2^ × 4.70 × 4.27 × 5.89^4^)^1/8^ = 5.13 eV

Therefore, we used the calculated $\chi$ of 5.13 eV to evaluate the band alignment. In addition, we determined the Fermi energy level ($E_{F}$) of the individual semiconductors by calculating their work function. The $E_{F}$ relates to the work function ($\Phi$) and the electrostatic potential energy in vacuum ($E_{\mathrm{vac}}$) as the following equation

$\Phi=E_{\mathrm{vac}}-E_{F}$.

The most stable surface terminations of Domains 3 and 4 (**Figure S1**) were taken to calculate the work functions using PBE and HSE06 functionals, as depicted in **Figure S4**. Finally, the band alignment of individual Domain 3 and 4 surfaces were obtained as illustrated in **Figure S5** where $E_{\mathrm{VBM}}$ and $E_{\mathrm{CBM}}$ were aligned versus vacuum level.

**Figure S4** Calculated in-plane averaged electrostatic potential along *z* axis of **a,** Domain3(112) and **b,** Domain4(1$\bar{1}$0) at the PBE and HSE06 level of theory, where $E_{\mathrm{vac}}$, $E_{F}$, and $\Phi$ represent vacuum energy, Fermi energy, and work function, respectively.

**Figure S5** Band alignment of individual Domain 3 and 4 surfaces at PBE and HSE06 level of theory, where $E_{F}$, $\Phi$, VBM, and CBM represent Fermi energy, work function, valence band maximum, and conduction band minimum, respectively. Note that PBE predicts a metallic character (zero band gap) for Domain 3 and 4, we therefore used the band gap values of bulk Domain 3 (1.10 eV) and Domain 4 (1.16 eV) calculated by HSE06 to construct the band alignments for all considered cases.

**Figure S6** Band alignment of bulk Domain 3 and 4 at the HSE06 level of theory, where $E_{g}$, $E_{F}$, VBM, and CBM represent band gap energy, Fermi energy, valence band maximum, and conduction band minimum, respectively.

# **Section S6:** **Calculation of optical absorption coefficient**

For semiconductor nanocrystals (NCs), understanding their chemical structure and morphology-dependent optical properties is crucial, both from a practical and a fundamental perspective. Multi-element chalcogenides NCs such as Cu_α_Zn_β_Sn_γ_Se_δ_ (CZTSe) exhibit unique optical effects depending on their shape, phase, and chemical composition. As reported in the previous experimental work, the photoluminescence (PL) spectra of different CZTSe NCs indicate that the emission-peak shape and position of CZTSe hexagonal WZ NCs, CZTSe quasi-tetrahedron ZB NCs, CZTSe ellipsoidal NCs, and CZTSe tetrapod NCs are different and CZTSe tetrapod NCs exhibit the highest PL intensity compared to others because of the combination of different phases.^[14]^ Moreover, it has been reported that the absorption coefficient is dependent on the size and volume of the NCs.^[15]^ Herein, to obtain a fundamental understanding of the optical properties of CZTSe tetrapod NCs, the frequency-dependent dielectric functions consisting of real and imaginary parts were examined to obtain the absorption coefficients of the deciphered Domain 3 and 4 in tetrapod structure. After the electronic ground state has been determined, the energy-dependent dielectric function was calculated. The imaginary part of dielectric function, $\varepsilon_{2}(\omega)$ is determined by the summation over empty conduction band states using the following expression,^[16]^

$$\varepsilon_{2}\left( \omega\right)=\frac{4\pi^{2}e^{2}}{\Omega}\lim_{q\to0} \frac{1}{q^{2}}\sum_{c,v,k} 2\omega_{k}\delta(\epsilon_{ck}-\epsilon_{vk}-\omega)\left\langle u_{ck+e_{\alpha}q} | u_{vk} \right\rangle\left\langle u_{ck+e_{\beta}q} | u_{vk} \right\rangle^{*}$$

where $\Omega$ is the volume of the primitive cell; $q$ is the electron momentum operator; $c$ and $v$ are the conduction and the valence band states, respectively; $\omega_{k}$ is the k-point weight; $\epsilon_{ck}$ and $\epsilon_{vk}$ are the energy eigenvalues of conduction and valence band at k-point, respectively; $u_{ck}$ and $u_{vk}$ are the cell periodic part wave functions of conduction and valence band at k-point, respectively; $e_{\alpha}$ and $e_{\beta}$ are the unit vectors along three directions, where $\alpha$ and $\beta$ are the Cartesian components. The real part of the dielectric function, $\varepsilon_{1}(\omega)$ is obtained by the Kramers-Kronig transformation from the following equation,^[16]^

$$\varepsilon_{1}\left( \omega\right)=1+\frac{2}{\pi}P\int_{0}^{\infty} \frac{\varepsilon_{2}\left( \omega' \right)\omega'}{{\omega'}^{2}-\omega^{2}}d\omega'$$

where P denotes the principal part of the integral. Finally, the optical absorption coefficient is calculated by the following relation,^[17]^

$\alpha\left( \omega\right)=\sqrt{2}\frac{\omega}{c}\left[ \sqrt{{\varepsilon_{1}}^{2}\left( \omega\right)+{\varepsilon_{2}}^{2}\left( \omega\right)}-\varepsilon_{1}(\omega) \right]^{1/2}$.

**Figure S7 a,** Optical absorption spectra of Domains 3 and 4. **b,** and **c,** The corresponding band structures of Domains 3 and 4, respectively. T_1_, T_2_, T_3_, and T_4_, represent the transition peaks 1, 2, 3, and 4, respectively.

# **References:**

[1] H. Ren, Y. Sun, F. Hoffmann, M. Vandichel, T. E. Adegoke, N. Liu, C. McCarthy, P. Gao, K. M. Ryan, *Nano Letters* **2024**, 24, 2125.

[2] a)G. Kresse, J. Furthmüller, *Physical Review B* **1996**, 54, 11169; b)G. Kresse, J. Furthmüller, *Computational Materials Science* **1996**, 6, 15; c)G. Kresse, J. Hafner, *Physical Review B* **1993**, 47, 558.

[3] a)P. E. Blöchl, *Phys. Rev. B* **1994**, 50, 17953; b)G. Kresse, D. Joubert, *Phys. Rev. B* **1999**, 59, 1758.

[4] J. Heyd, G. E. Scuseria, M. Ernzerhof, *The Journal of Chemical Physics* **2003**, 118, 8207.

[5] a)M. Han, X. Zhang, Z. Zeng, *Physical Chemistry Chemical Physics* **2017**, 19, 17799; b)D. Han, Y. Sun, J. Bang, Y. Zhang, H.-B. Sun, X.-B. Li, S. Zhang, *Physical Review B* **2013**, 87, 155206; c)J. Pohl, K. Albe, *Physical Review B* **2013**, 87, 245203.

[6] T. M. Henderson, J. Paier, G. E. Scuseria, *physica status solidi (b)* **2011**, 248, 767.

[7] a)C. G. Broyden, *IMA Journal of Applied Mathematics* **1970**, 6, 76; b)C. G. Broyden, *IMA journal of applied mathematics* **1970**, 6, 222.

[8] J. J. Jorgensen, G. L. Hart, *Modelling and Simulation in Materials Science and Engineering* **2021**, 29, 065014.

[9] H. J. Monkhorst, J. D. Pack, *Physical Review B* **1976**, 13, 5188.

[10] a)M. Mousavi, A. Habibi-Yangjeh, *Advanced Powder Technology* **2018**, 29, 94; b)A. Raza, A. A. Haidry, J. Saddique, *Journal of Physics and Chemistry of Solids* **2022**, 165, 110694; c)C. Khamdang, S. Singsen, A. Ngoipala, I. Fongkaew, A. Junkaew, S. Suthirakun, *ACS Applied Energy Materials* **2022**, 5, 13997.

[11] S. R. Morrison, S. Morrison, *Electrochemistry at semiconductor and oxidized metal electrodes*, Vol. 126, Springer, **1980**.

[12] a)Q. Yuan, L. Chen, M. Xiong, J. He, S.-L. Luo, C.-T. Au, S.-F. Yin, *Chemical Engineering Journal* **2014**, 255, 394; b)M. Mousavi, A. Habibi-Yangjeh, M. Abitorabi, *Journal of colloid and interface science* **2016**, 480, 218.

[13] S. Kim, J. Chen, T. Cheng, A. Gindulyte, J. He, S. He, Q. Li, B. A. Shoemaker, P. A. Thiessen, B. Yu, *Nucleic Acids Research* **2023**, 51, D1373.

[14] H. Ren, Z. Li, Y. Sun, P. Gao, C. McCarthy, N. Liu, H. Xu, K. M. Ryan, *Chemistry of Materials* **2020**, 32, 7254.

[15] a)A. Salant, M. Shalom, Z. Tachan, S. Buhbut, A. Zaban, U. Banin, *Nano Letters* **2012**, 12, 2095; b)J. L. Peters, J. de Wit, D. l. Vanmaekelbergh, *Chemistry of Materials* **2019**, 31, 1672.

[16] M. Gajdoš, K. Hummer, G. Kresse, J. Furthmüller, F. Bechstedt, *Phys. Rev. B* **2006**, 73, 045112.

[17] X. Niu, Y. Li, H. Shu, X. Yao, J. Wang, *The Journal of Physical Chemistry C* **2017**, 121, 3648.
